# Endophytic bacterial community of grapevine leaves influenced by sampling date and phytoplasma infection process

**DOI:** 10.1186/1471-2180-14-198

**Published:** 2014-07-21

**Authors:** Daniela Bulgari, Paola Casati, Fabio Quaglino, Piero A Bianco

**Affiliations:** 1 Dipartimento di Scienze Agrarie e Ambientali-Produzione, Territorio, Agroenergia, Università degli Studi, via Celoria 2, 20133 Milan, Italy

**Keywords:** Flavescence dorée, Recovery, Taxon-specific real-time PCR, LH-PCR, Microbial ecology

## Abstract

**Background:**

Endophytic bacteria benefit host plant directly or indirectly, e.g. by biocontrol of the pathogens. Up to now, their interactions with the host and with other microorganisms are poorly understood. Consequently, a crucial step for improving the knowledge of those relationships is to determine if pathogens or plant growing season influence endophytic bacterial diversity and dynamic.

**Results:**

Four healthy, four phytoplasma diseased and four recovered (symptomatic plants that spontaneously regain a healthy condition) grapevine plants were sampled monthly from June to October 2010 in a vineyard in north-western Italy. Metagenomic DNA was extracted from sterilized leaves and the endophytic bacterial community dynamic and diversity were analyzed by taxon specific real-time PCR, Length-Heterogeneity PCR and genus-specific PCR. These analyses revealed that both sampling date and phytoplasma infection influenced the endophytic bacterial composition. Interestingly, in June, when the plants are symptomless and the pathogen is undetectable (i) the endophytic bacterial community associated with diseased grapevines was different from those in the other sampling dates, when the phytoplasmas are detectable inside samples; (ii) the microbial community associated with recovered plants differs from that living inside healthy and diseased plants. Interestingly, LH-PCR database identified bacteria previously reported as biocontrol agents in the examined grapevines. Of these, *Burkholderia, Methylobacterium* and *Pantoea* dynamic was influenced by the phytoplasma infection process and seasonality.

**Conclusion:**

Results indicated that endophytic bacterial community composition in grapevine is correlated to both phytoplasma infection and sampling date. For the first time, data underlined that, in diseased plants, the pathogen infection process can decrease the impact of seasonality on community dynamic. Moreover, based on experimental evidences, it was reasonable to hypothesize that after recovery the restructured microbial community could maintain the main structure between seasons.

## Background

A multiplicity of definitions has been applied to the term ‘endophyte’ with different interpretations. In accordance with Schulz and Boyle [1], endophytic bacteria are bacteria that live inside a plant without causing apparent diseases. They have been isolated from a wide range of plants ranging from monocotyledonous to dicotyledonous [among others [2-5]]. Endophytes often originate from the soil, initially infecting the host plant by colonizing, for instance, the cracks formed in lateral root junctions and then quickly spreading to the spaces in the root. Moreover, bacteria can gain entry the interior part of plant through stomata, epidermal junctions, stem and flowers [6,7]. Once inside the plant, bacteria remain localized in a specific tissue, such as the root cortex, or colonize the plant systematically by transport or active migration through the conducting elements. Some endophytic bacteria seem to positively influence plant-host growth through similar mechanisms described for plant-growth promoting rhizobacteria (PGPR) [8]. Moreover, they can promote plant growth by reducing the deleterious effects of plant pathogens through direct or indirect mechanisms (biocontrol) [9]. Bacteria can directly antagonize pathogens by competition for root niches or by producing allelochemicals (siderophores, antibiotics, biocidal, lytic enzymes, and detoxification enzymes), by pathogen virulence factors degradation or by the interference with pathogens quorum-sensing [8].

At the beginning, endophytes were isolated on growth culture media after surface disinfection of different plant tissues. Bacteria species isolated with cultivation-dependent methods are selected by cultivation media, growth conditions (different from those present in the host plant: lack of obligate endophytes) and plant tissue manipulation [10]. The employment of cultivation-independent fingerprinting molecular methods based on 16S rRNA gene analyses allowed a more specific, replicable and detailed description of microbial diversity. In detail, Length Heterogeneity-PCR and taxon specific real-time PCR were used for studying endophytic microbial ecology in different environments. Recently, these techniques have been applied for investigating how endophytic community composition is influenced by different parameters, such as plant genotype, growth stage, physiological status, tissue, environmental conditions and agricultural practices. Furthermore, the effects of pathogens on endophytic bacterial communities associated with plants have been examined [5,11,12]. Interestingly, LH-PCR analyses highlighted that phytoplasmas can restructure the endophytic bacterial community associated with grapevines by selecting bacterial strains that could elicite plant defense response leading to recovery (spontaneous remission of symptoms and turning back to healthy condition) from Flavescence dorée, a disease of the grapevine yellows complex [13].

Up to now, few studies investigated how sampling date and pathogen infection, two parameters whose effects could be overlapped and not easily distinguishable, shape endophytic bacterial community [11,12,14].

With this aim, we studied leaf endophytic bacteria in healthy, phytoplasma-diseased and recovered grapevine plants sampled from June to October 2010. The bacterial community structure and diversity were investigated by taxon-specific real-time PCR and LH-PCR. These assays showed that both phytoplasma infection and sampling date shaped the endophytic bacterial community.

## Methods

### Grapevine samples collection and DNA extraction

In 2010, grapevine leaf samples were collected in a vineyard in Lombardy region (north-western Italy) on the basis of previous grapevine yellows (GY) survey evidencing the spreading of Flavescence dorée phytoplasma (FDp) [15]. Four healthy, four FDp-diseased and four recovered *Vitis vinifera* cv. Barbera plants were selected and sampled each month from June (no expression of phytoplasma related symptoms) to October (severe expression of phytoplasma related symptoms) (Table 1). Moreover, climatic parameters (temperature, humidity, rainfall and wind) for each sampling month were downloaded from ARPA (Regional Agency for Environmental Protection) station, located in Voghera (PV), Lombardy region (Table 2).

**Table 1:** Phytoplasma quantification in grapevine samples collected in different months

**Sample ID**	**Conditions**	**No. *rplN *** **gene molecole/ng of total DNA**				
		**June**	**July**	**August**	**September**	**October**
1	asymptomatic	-	-	-	-	-
2	asymptomatic	-	-	-	-	-
3	asymptomatic	-	-	-	-	-
4	asymptomatic	-	-	-	-	-
5	symptomatic	-	17.64	28.19	211.21	351.2
6	symptomatic	-	-	-	0.35	1.45
7	symptomatic	-	20.5	-	17.9	22.45
8	symptomatic	-	25.9	157.08	1.53	0.15
9	recovered	-	-	-	-	-
10	recovered	-	-	-	-	-
11	recovered	-	-	-	-	-
12	recovered	-	-	-	-	-

**Table 2:** Climatic parameters recorded by regional service during sampling month

**Month**	**Mean T**	**Value min T**	**Value max T**	**Total rainfall**	**Humidity**	**Wind**
June	22.4°C	13.1°C	33.8°C	62.8 mm	51%	15 Km/h
July	26.6°C	15.0°C	36.0°C	1.2 mm	51%	8 Km/h
August	23.4°C	13.2°C	32.1°C	66.2 mm	59%	9 Km/h
September	18.3°C	9.6°C	28.3°C	31.2 mm	66%	9 Km/h
October	13.6°C	4.4°C	23.8°C	78.4 mm	82%	13 Km/h

Symptomatic plants showed typical grapevine yellows symptoms including desiccation of inflorescences, berry shrivel, leaf discolorations, reduction of growth and irregular ripening of wood [16]. Recovered grapevine plants have not shown GY symptoms since 2003. Leaf tissues preparation and total DNA extraction from 20 g of grapevine leaf were carried out as previously described [13].

### Phytoplasma detection and quantification by real-time PCR

Molecular identification and FDp quantification were carried out by real-time PCR on 25 ng of total DNA extracted from each sample. Reactions were performed using the commercial kit Real-time PCR (TaqMan) (IPADLAB, Lodi, Italy). Each sample was amplified in duplicate. Thermocycling was carried out on the 7300 Real Time PCR System (Applied Biosystems), and consisted of an initial denaturation at 95°C for 10 min followed by 40 cycles of 15 s at 95°C and 1 min at 60°C [17]. The target gene sequence (gene *rplN*, coding the ribosomal protein L14) of FDp was PCR amplified and cloned into pCR2.1 (Invitrogen) for use as standard template. The assay values were obtained with the Standard Curve Method using serially diluted standard template (from 10^6^ to 1 copy of target gene) in total DNA extracted from healthy grapevine plants. Amplification efficiency in each PCR assay was calculated by 10^-1/slope^, where slope was obtained from the plot of log transformation of serial diluted target copy number versus threshold cycle. Diluted standard templates were analyzed in triplicate for standard curve construction.

### Taxon-specific real-time PCR

*Actinobacteria*, *Firmicutes*, *Gammaproteobacteria*, and *Alfaproteobacteria* were detected and quantified by taxon-specific real-time PCR. PCR reactions were performed as previously described by Fierer *et al*. [18] and Bacchetti de Gregoris *et al*. [19]. Primers sequence and specificity are reported in Table 3. Each sample was amplified in duplicate. Thermocycling was carried out on the 7300 Real Time PCR System (Applied Biosystems), and consisted of an initial denaturation at 95°C for 10 min followed by 40 cycles of 15 s at 95°C and 1 min at 60°C. The target gene sequence (16S rRNA) of taxon representative bacteria were PCR amplified and cloned into pGEM®-T easy vector (Promega) for use as standard template. The assay values were obtained with the Standard Curve Method using serially diluted standard template (from 10^8^ to 10^1^ copy of target gene). Amplification efficiency in each PCR assay was calculated by 10^-1/slope^, where slope was obtained from the plot of log transformation of serial diluted target copy number versus threshold cycle. Diluted standard templates were analyzed in triplicate for standard curve construction.

**Table 3:** Nucleotide sequences and specificity of primers employed in the present study

**Name**	**Primer sequence (5’-3’)**	**Gene target**	**Taxon target**	**Reference**
6S-27F	AGAGTTTGATCCTGGCTCAG	*16S rRNA*	Bacteria	[19]
338R	GCTGCCTCCCGTAGGAGT	*16S rRNA*	Bacteria	[19]
α682F	CIAGTGTAGAGGTGAAATT	*16S rRNA*	*α-Proteobacteria*	[18]
908αR	CCCCGTCAATTCCTTTGAGTT	*16S rRNA*	*α-Proteobacteria*	[18]
1080γF	TCGTCAGCTCGTGTYGTGA	*16S rRNA*	*γ-Proteobacteria*	[18]
γ1202R	CGTAAGGGCCATGATG	*16S rRNA*	*γ-Proteobacteria*	[18]
Act920F3	TACGGCCGCAAGGCTA	*16S rRNA*	*Actinobacteria*	[18]
Act1200R	TCRTCCCCACCTTCCTCCG	*16S rRNA*	*Actinobacteria*	[18]
Lgc353	GCAGTAGGGAATCTTCCG	*16S rRNA*	*Firmicutes*	[17]
Eub518	ATTACCGCGGCTGCTGG	*16S rRNA*	*Firmicutes*	[17]
MB4	CCGCGTGAGTGATGAAGG	*16S rRNA*	*Methylobacterium*	[21]
MB	AGCGCCGTCGGGTAAGA	*16S rRNA*	*Methylobacterium*	[21]
Sph-spt 694f	GAGATCGTCCGCTTCCGC	*spt*	*Sphingomonas*	[22]
Sph-spt 983r	CCGACCGATTTGGAGAAG	*spt*	*Sphingomonas*	[22]
Gro1	CTGGAAGACATCGCGATC	*groEL*	*Burkholderia*	[20]
Gro2	CGTCGATGATCGTCGTGTT	*groEL*	*Burkholderia*	[20]
pagF	CACTGGAAACGGTGGCTAAT	*16S rRNA*	*Pantoea*	[23]
pagR	CGGCAGTCTCCTTTGAGTTC	*16S rRNA*	*Pantoea*	[23]

### LH-PCR profiles associated with grapevine samples

To study endophytic bacterial structure and diversity from June to October in healthy, FDp-diseased and recovered grapevine plants, total DNA was analyzed by Length Heterogeneity-PCR (LH-PCR). The LH-PCR reaction was done with the primers 27F labelled at its 5’ end with the phosphoramidite dye (6-FAM) and 338R as previously described [13]. Primers sequence and specificity are reported in Table 3. Quantified PCR products (25 ng) were added to 0.8 μl of 500 ROX-labelled internal size standard and 15 μl of deionized formamide (Applied Biosystems, Italy). Samples were denatured at 95°C for 8 min, rapidly put into ice for 5 min, and loaded on the ABI Prism 310 as described in Brusetti *et al*. [20]. LH-PCR data were analyzed with Genescan 3.1.2 software (Applied Biosystems), and a threshold of 50 fluorescent units was used. Peak sizing and peak matrix were done with the Genescan 3.1.2 software. The position of all peaks was carefully checked by eye. For all grapevine samples PCR amplification was run three times and three separate PCRs were also run to confirm the LH-PCR peak sizing through different PCR reactions.

### Statistical analyses

Statistical analyses were carried out, independently for each bacterial group, to study the variation of endophytic bacterial composition in association with phytoplasma infection process and seasonality. qPCR data were processed by two-way ANOVA (α = 0.05) in order to evaluate the combined influence of sanitary status of the plants and seasonality (interaction of the two variables) on quantitative fluctuation of single taxa in the samples analyzed. In the case of no interaction, Duncan test was applied to investigate separately the influence of each variable on qPCR results; otherwise, Sidak test was employed. qPCR data were processed by the use of the software SPSS (Statistical Product and Service Solutions, IBM statistics 19).

Profiles obtained by LH-PCR analysis of healthy, diseased and recovered grapevines samples were processed together by correspondence analysis (CA) in order to evaluate the combined effect of phytoplasma infection and sampling date. The same analysis was repeated separately on healthy and recovered grapevine plants collected from June to October in order to specifically investigate the seasonality influence. Furthermore, CA was applied to observe possible associations among phytoplasma infection process and microbial composition variation on LH-PCR profiles obtained from diseased grapevine collected from June to October.

The statistics were performed with JMP software (JMP, version 7, SAS Institute Inc., Cary, NC, 1989–2007).

### Genus specific PCR

On the basis LH-PCR database and taxon-specific real-time PCR results, the presence of different genera were analyzed during phytoplasma infection and growing season. In detail, the genera *Burkholderia, Methylobacterium, Sphingomonas*, and *Pantoea* were detected by PCR as previously described respectively by Suppiah *et al*. [21], Podolich *et al*. [22], Yim *et al*. [23], and Vorwerk *et al*. [24]. Primers sequence and specificity are reported in Table 3.

## Results and discussion

### Phytoplasmas detection and quantification

TaqMan real-time assay was carried out in order to detect and quantify phytoplasmas in the analyzed samples. The consistency of the real-time PCR assay was confirmed by the strong linear inverse relationship between the threshold cycle numbers and the copy numbers of *rplN* phytoplasma gene for primer sets (R^2^ = 0.99). The slope value was -3.1 indicating that the amplification efficiency was more than 99%. No phytoplasma was detected in all grapevine plants sampled in June. On the contrary, symptomatic plants from July to October were characterized by the presence of phytoplasmas, except for samples n. 6 and n. 7 where phytoplasmas were not detected in August probably due to their sporadic distribution in plant tissues [25]. Phytoplasma concentration was calculated on the basis of standard curve and it was expressed as phytoplasma molecules on ng of total DNA extracted (Table 1). In symptomatic plants the phytoplasma concentration was influenced by the grapevine sample but it increased during the season reaching the highest titer in October when the symptoms were severe, confirming evidence from other study [26]. As previously reported [27], no phytoplasmas were detected in healthy and recovered plants.

### Taxon specific real-time PCR

Quantitative PCR (qPCR) was employed for studying the microbial community abundance in several complex environments, e.g. marine water, lake water, soil sediments and plants [28]. qPCR was also applied to asses soil and plant microbial community structure at broad taxonomic level [12,18]. In this study, we applied taxon specific real-time PCR in order to evaluate quantitatively the influence of sampling date and phytoplasma infection on different bacterial groups. In detail, *Alfaproteobacteria*, *Gammaproteobacteria, Actinobacteria*, and *Firmicutes* were chosen on the basis of previous studies describing the endophytic bacterial community associated with grapevine plants [13,29].

Two-way ANOVA test showed that *Alfaproteobacteria*, *Gammaproteobacteria*, and *Firmicutes* abundance was not influenced by the interaction between sanitary status (healthy, diseased, and recovered) of the plants and sampling date. This result demonstrates that the two variables are independent. Duncan test revealed that taxon-abundance for these bacterial groups was influenced by sampling date (Table 4). In detail, *Alfaproteobacteria* and *Gammaproteobacteria* quantity was significantly different in August and June respectively, and in these months were registered the highest concentration (Table 4). *Gammaproteobacteria* members exhibit broad range of temperature adaptation [30] and in June higher variability of this parameter was registered (Table 2). Moreover, qPCR data from *Firmicutes* varied according to the sampling date into three clusters: June/October, July/August and September. One can speculate that maximum temperature recorded in July and August could decrease the abundance of some endophytic bacteria, while mild temperatures and high humidity registered in September could be related to its increase. Different studies reported that the endophytic bacterial community dynamics were influenced by season warming and plant development [14,31]. Our data are in agreement with these studies but also revealed that sampling date influence quantitatively the bacterial community at high taxonomic levels.

**Table 4:** Means of 16S rDNA molecules for month in homogeneous subset based on real-time data analyzed by Duncan test

**Bacterial group**	Month^*^	**No. of plants**	**Subset**	
			**1**	**2**
*Alfaproteobacteria*^a,b^	October	12	4.25	
	June	11	46.64	
	September	12	52.50	
	July	11	55.82	
	August	12		136.50
	*Significance*		*0.173*	*1.000*
*Gammaproteobacteria*^c,b^	September	12	25895.00	
	July	12	41989.83	
	October	12	52245.75	
	August	12	76307.75	
	June	11		2109394.27
	*Significance*		*0.825*	*1.000*
*Firmicutes*^d,b^	October	12	2044.50	
	June	11	2396.91	
	July	11	3873.36	3873.36
	August	12	4107.25	4107.25
	September	12		5026.75
	*Significance*		*0.061*	*0.278*

^*^Months were in order of size; ^a^Uses harmonic mean sample size = 11,579; ^b^α = 0.05; ^c^Uses harmonic mean sample size = 11,786; ^d^Uses harmonic mean sample = 11,579.

**Figure 1:**
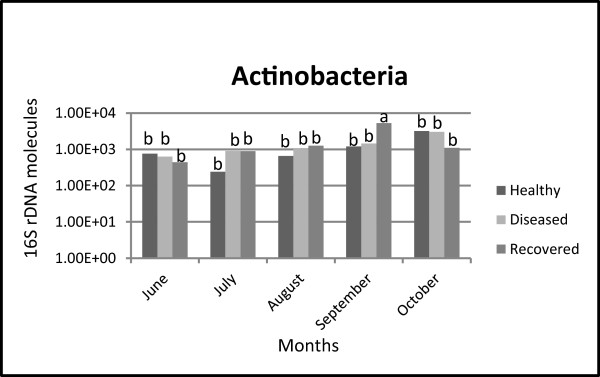
***Actinobacteria-*specific real-time PCR data analysed by Sidak test.** Presence and distribution of *Actinobacteria* were examined in healthy, diseased and recovered grapevines sampled from June to October. Within the graphic, bars marked with different letters are significantly different (*P* < 0.05).

On the other hand, *Actinobacteria* abundance was correlated with the interaction between sanitary status of the plants and sampling date. Sidak test showed that abundance of *Actinobacteria* associated with recovered plants in September was distinguished from (i) recovered plants collected in all the other months, and (ii) healthy and diseased plants collected in September (Figure 1 and Additional file 1). The endophytic bacterial community can be influenced by the presence of phytopathogens [32,33]. In fact, Trivedi and colleagues [11] reported that pathogen infection restructured the endophytic bacterial community quantitatively. Moreover, in previous studies we reported that phytoplasma infection influences qualitatively the microbial community composition [5,13]. In detail, the comparison between the endophytic bacterial community associated with healthy and phytoplasma infected apple trees showed differences at species level. The qPCR approach adopted in the present study was at broad level of taxonomic resolution, which would mask the diversity at species level [18].

### General analyses of LH-PCR profiles in healthy, diseased and recovered grapevine plants

**Figure 2:**
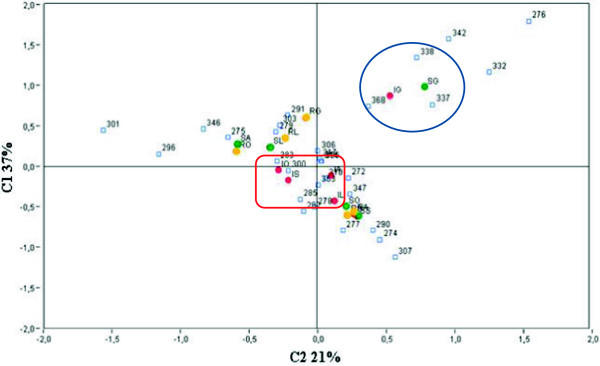
**Correspondence analyses of overall LH-PCR profiles.** LH-PCR data statistically analysed by correspondence analyses (CA). The two axes represent the percent variation in species. Two different clusters were present in CA diagram. Healthy and diseased grapevine plants sampled in June, both coordinates positive grouped at the right of the diagram (graphically represented inside the blue circle); results of diseased plants sampled from July to October clustered around the centre of the axis (graphically represented inside the red square). Healthy, recovered and diseased grapevine plants collected from June to October are represented by green, yellow and red dots, respectively. S = healthy plants; I = diseased plants; R = recovered plants; G = June; L = July; A = August; S = September; O = October. Within the graphics, numbers represent the peak size.

In order to investigate the endophytic bacterial community dynamics at lower taxonomic level (genus level), LH-PCR was performed on sixty grapevine samples. LH-PCR data of the sixty samples were processed by correspondence analysis (CA) and graphically portrayed (Figure 2). We decided to process all the data together in order to evaluate the influence of sampling date and phytoplasma infection on the bacterial community composition. In detail, the ordination axes represent the percentage of variation in microbial composition associated with the samples analysed. Figure 2 showed that endophytic bacterial community associated with healthy and diseased plants sampled in June differed along C1 and C2 from that which lived in association with healthy, diseased and recovered plants collected in all the other months. Interestingly, diseased plants sampled in June were asymptomatic and PCR negative suggesting that, before phytoplasmas replication inside plant tissues, the endophytic bacterial community associated with diseased plants was similar to healthy ones. On the other hand, recovered grapevine plants sampled in June were not significantly different from the analysed samples collected throughout the season. Although recovered plants sampled in June, as well as healthy and diseased plants, were asymptomatic and PCR negative, their LH-PCR data did not cluster together. In a previous work, beneficial microbes noticed as systemic resistance inducers (ISR-inducers) have been found in association with recovered grapevine plants [13]. ISR could result in a decrease in plant susceptibility and disease severity or as a reduction in the number of diseased plants [34].

Moreover, it was hypothesized that alterations induced by phytoplasmas in the grapevine endophytic bacterial community select bacterial strains that are more resistant to ROS and able to elicit plant defense responses, including ROS as well; these bacteria could ultimately lead to recovery. This view is supported by previously reported findings showing that recovered grapevine plants have higher levels of ROS than those of diseased and healthy plants [35]. In this work LH-PCR data suggested that after recovery the restructured microbial community could maintain the main structure between seasons suggesting a possible role of endophytes in protecting plant from re-infection events.

Moreover, diseased plants collected from July to October clustered around the centre of the axes indicating a lower variation of bacterial composition during the season. On the contrary, healthy and recovered plants showed a possible endophytic bacterial community variation during the season.

### LH-PCR data related to sampling date

**Figure 3:**
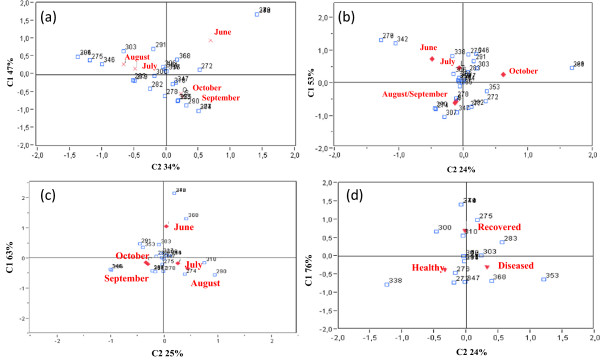
**Correspondence analyses of LH-PCR profiles based on sanitary condition and sampling date.** LH-PCR profiles of **(a)** healthy, **(b)** recovered and **(c)** diseased grapevine plants sampled throughout the season and of **(d)** healthy, diseased and recovered grapevine plants sampled in June analyzed by correspondence analyses. Within the graphics, numbers represent the peak size.

Correspondence analysis was repeated separately on healthy and recovered grapevine plants collected monthly from June to October. This analysis made it possible to evaluate the influence of sampling date on endophytic bacterial community composition.Significant clustering (P ≤ 0.05) was observed on the basis of sampling dates. In detail, the higher diversity in both cases was explained by C1.This component explicated the stronger diversity between the samples collected in June in comparison with those collected in September/October (healthy plants) and August/September (recovered plants). Moreover, the healthy grapevines collected in July/August and in September/October were portrayed in two different clusters in the CA graph (Figure 3a and b). In the case of recovered plants, samples collected in October were significantly separated (C2) from all the others (Figure 3b). The influence of sampling date on endophyte composition displayed by taxon specific real-time PCR was also confirmed at genus level by LH-PCR analyses.

### LH-PCR data related to phytoplasma infection

In order to evaluate the endophytic bacterial dynamics during phytoplasma infection process, LH-PCR data carried out on phytoplasma infected grapevine sampled monthly from June to October were processed by CA.Figure 3c showed that the bacterial community associated with diseased plants in June (negative for phytoplasma detection) was different in comparison to the same plants sampled in the other months. This clustering was represented by the C1 (63%) that explained the higher diversity among samples. On the contrary the C2 explained only the 25% of diversity indicating that grapevines sampled in the other months shared a higher number of LH-PCR profiles.

In order to clarify the effect of phytoplasma presence on microbial composition, CA was repeated only on healthy, diseased and recovered plants collected in June, when the phytoplasma replication in infected plants was not detectable. Intriguingly, the analysis highlighted that bacterial communities of diseased (FDp still not detectable) and healthy plants in June were significantly similar (C2 24%) and strongly differed from those associated with recovered plants (C1 76%) (Figure 3d). Some studies reported that phytopathogens affected the structure of plant-associated bacterial community [5,11,31,32,36,37]. In accordance with the literature, our data showed that there is a correlation between phytoplasma replication and microbial diversity associated with diseased plants and, for the first time, revealed that the pathogen infection process can decrease the impact of seasonality on community dynamic.

Recently, Trivedi and colleagues [12] showed that ‘*Candicatus* Liberibacter asiaticus’ determined a restructuring of rhizosphere microbial communities and induced also a change in their functional potential. In our case, it could be interesting to study the functional diversity of endophytic bacterial community associated with diseased and recovered plants in order to clarify the relation between phytoplasmas and endophytic bacterial community activity.

### Endophytic bacteria detection by LH-PCR

To identify bacterial species associated with LH-PCR peaks we compared the results of LH-PCR analysis carried out on the metagenome of the examined plants with the LH-PCR database previously published [4,13]. LH-PCR analysis allowed detection of a total of 30 peaks in the samples analysed in the present study. Unfortunately, the comparison with LH-PCR database registered several non-identified peaks, indicating that the diversity of endophytic bacteria in grapevine leaves is higher than that described until now [28,38]. This highlights the bias inherent to any molecular technique that uses the PCR-based 16S rRNA gene sequence analyses for microbial community description [39]. However LH-PCR has been successfully utilized to characterize the bacterial community associated with different environments, including plants [4,20,40,41]. In present work only eight peaks were attributed to the correspondent bacteria by comparison with the LH-PCR database. In detail, *Methylobacterium gregans*/*Sphingomonas* sp. (peak at 314 bp), *Burkholderia fungorum*/Chloroplast (peak at 317 bp) and *Bacillus* sp. (peak at 356 bp) were detected in all plants analysed and in all months. This data showed that these bacteria were constantly associated with grapevine plants characterized by different health condition. On the contrary, the presence of other bacteria was influenced by the sampling date or by the sanitary status of the plants (Table 5). In detail, a bacterium belonging to the family *Sphingomonandaeae* (peak at 310 bp) was constantly associated with recovered grapevine plants from June to October, while in healthy and diseased plants it disappeared respectively in August and September. Furthermore, *Burkholderia* sp./*Paenibacillus pasadenensis* (peak at 338 bp) was not detected in diseased plants but only in healthy plants in June and in recovered plants in July. *Pectobacterium* sp. (peak at 342 bp) was mainly associated with healthy, diseased and recovered grapevines in June. *Ewingella americana* (peak at 346 bp) was detected only in few grapevines but it has two peaks of presence in the recovered grapevines sampled in June and in October. Moreover, *Pantoea agglomerans* (peak at 347 bp) was constantly associated with healthy and diseased plants during the season, while in recovered grapevines its presence was detected from August to October. These data highlighted a bacterial fluctuation during the growing season and in detail, the bacterial diversity seems to be higher in the last growing season (October) than in early stages (June). This evidence is in agreement with previous works showing that plant developmental stage influences the endophytic bacterial composition increasing the endophytes population at lower temperature indicating the ability of endophytes to change their metabolism [31].

**Table 5:** Presence of LH-PCR peaks attributed to endophytic bacteria in grapevine plants over the season

**Plant**^**a**^	**Peak**																								
	**310 bp**^**b**^					**342 bp**^**c**^					**338 bp**^**d**^					**346 bp**^**e**^					**347 bp**^**f**^				
	**Jun**	**Jul**	**Aug**	**Sept**	**Oct**	**Jun**	**Jul**	**Aug**	**Sept**	**Oct**	**Jun**	**Jul**	**Aug**	**Sept**	**Oct**	**Jun**	**Jul**	**Aug**	**Sept**	**Oct**	**Jun**	**Jul**	**Aug**	**Sept**	**Oct**
1 h	-	-	-	-	+	+	-	-	-	-	-	-	-	-	-	-	+	-	-	-	-	-	-	+	+
2 h	-	+	-	+	-	+	-	-	-	-	-	-	-	-	-	-	-	-	-	-	-	+	-	+	+
3 h	+	+	-	-	+	+	-	-	-	-	-	-	-	-	-	-	-	-	-	-	+	+	+	+	+
4 h	-	+	-	+	+	+	-	-	-	-	+	-	-	-	-	-	-	-	-	-	+	-	+	+	+
1d	-	+	+	-	-	+	-	-	-	-	-	-	-	-	-	-	-	-	-	-	+	+	-	+	-
2d	-	+	+	-	-	+	-	-	-	-	-	-	-	-	-	-	-	-	-	-	+	-	+	+	+
3d	-	-	-	-	-	+	-	-	-	-	-	-	-	-	-	-	-	-	+	+	-	-	+	-	+
4d	+	+	-	-	+	+	-	-	-	-	-	-	-	-	-	-	-	-	-	-	-	+	+	+	+
1r	+	-	+	+	+	+	+	-	-	-	-	-	-	-	-	+	-	-	-	-	-	-	+	+	-
2r	+	+	-	+	-	+	-	-	-	-	-	+	-	-	-	+	-	-	-	-	-	-	+	+	+
3r	+	-	-	+	+	+	-	-	-	-	-	-	-	-	-	-	-	-	-	+	-	-	+	+	-
4r	-	+	+	+	+	+	-	-	-	-	-	-	-	-	-	-	-	-	-	+	-	-	+	+	-

^a^h: healthy grapevine plants; d: diseased grapevine plants; r: recovered grapevine plants.

^b^*Sphingomonadaceae* bacterium; ^c^*Pectobacterium* sp.; ^d^*Burkholderia* sp./*Paenibacillus pasadenensis*; ^e^*Ewingella americana*; ^f^*Pantoea agglomerans.*

### Genera fluctuation

Interestingly, *Burkholderia, Methylobacterium, Sphingomonas* and *Pantoea,* bacteria identified by LH-PCR database, have been reported in the literature as biocontrol agents, but their plant protection mechanisms are not well known [42-45]. As LH-PCR has some limitations such as the difficulty to resolve profiles due to the contiguous amplicon distributions and the length amplicon overlapping of bacteria belonging to phylogenetically distinct taxon [39], we decided to investigate more accurately the distribution of these genera by genus-specific PCR. In detail, *Sphingomonas* was found constantly associated with healthy, diseased and recovered grapevine plants collected from June to October. *Pantoea* was present in healthy and diseased plants from June to October while in recovered plants was detected only in July, August and October. These data confirmed the *Sphingomonas* and *Pantoea* identification carried out by LH-PCR. *Burkholderia* and *Methylobacterium* were not identified in June but they are detected in all the analysed plants in September. In detail, *Burkholderia* was detectable in healthy plants in the late season (September and October) while in diseased and recovered plants from July (Table 6). On the basis of these data, it is evident that *Burkholderia, Methylobacterium*, and *Pantoea* dynamics were influenced by the phytoplasma infection process and seasonality, while *Sphingomonas* distribution seems to be independent. To the best of our knowledge, it is the first report of *Burkholderia, Methylobacterium, Sphingomonas*, and *Pantoea* dynamics during phytoplasma infection process. Due to the different influence of phytoplasma infection process and seasonality on the dynamics of such bacteria, it should be intriguing to carried out further studies focused on determining the plant protection mechanisms utilized by those endophytic bacteria.

**Table 6:** Presence of bacterial genera reported as biocontrol agents in grapevine plants over the season

**Plant**^**a**^	**Genus**																			
	***Sphingomonas***					***Methylobacterium***					***Burkholderia***					***Pantoea***				
	**Jun**	**Jul**	**Aug**	**Sept**	**Oct**	**Jun**	**Jul**	**Aug**	**Sept**	**Oct**	**Jun**	**Jul**	**Aug**	**Sept**	**Oct**	**Jun**	**Jul**	**Aug**	**Sept**	**Oct**
1 h	+	-	-	+	+	-	-	+	+	+	-	-	-	+	-	+	-	+	+	+
2 h	+	-	-	+	+	-	+	+	+	+	-	-	-	+	+	+	+	-	-	+
3 h	+	+	+	+	+	-	-	+	+	+	-	-	-	+	-	+	+	+	-	-
4 h	-	-	-	-	+	-	-	+	+	-	-	-	-	+	-	+	+	+	+	+
1d	+	-	-	-	+	-	-	+	+	+	-	+	-	+	+	+	+	+	-	+
2d	+	+	+	+	+	-	+	+	+	+	-	-	+	+	+	+	+	+	-	+
3d	+	+	+	+	+	-	+	+	+	+	-	-	+	+	-	-	-	+	+	+
4d	+	+	+	+	+	-	+	+	+	+	-	-	+	+	-	-	-	+	+	+
1r	+	+	+	+	-	-	+	+	+	+	-	+	+	+	-	-	+	+	-	-
2r	+	+	+	+	+	-	+	-	+	+	-	+	+	+	+	-	+	+	-	-
3r	+	+	+	-	+	-	+	+	+	+	-	-	+	+	-	-	+	+	-	+
4r	+	+	+	+	+	-	+	-	+	+	-	+	+	+	-	-	+	-	-	+

^a^h: healthy grapevine plants; d: diseased grapevine plants; r: recovered grapevine plants.

## Conclusion

In this study, three different molecular techniques were employed for investigating the microbial community composition and fluctuation in grapevine highlighting that they are closely related to phytoplasma infection process and seasonality. At a broad taxonomic level, statistical analyses highlighted that the two parameters were independent and the sampling date shaped the dynamic of *Firmicutes*, *Gammaproteobacteria* and *Alfaproteobacteria*, while the *Actinobacteria* dynamic was correlated to the interaction of the two factors. Interestingly, at lower taxonomic level our data showed that phytoplasmas replication could alter the microbial diversity associated with diseased plants and, for the first time, evidenced that the pathogen infection process could decrease the impact of seasonality on community dynamic. Moreover, microbial community associated with recovered plants in June, when phytoplasma replication was not active, differs from that of healthy and diseased plants, suggesting that, after recovery, the restructured microbial community could maintain the main structure throughout the seasons. This data along with the presence of potential biocontrol agents in the examined grapevines suggest a possible role of endophytes in protecting plant from re-infection events. Future studies will be carried out for describing the role of microbial community in the shift from diseased condition to recovered one.

## Competing interests

The authors declare that they have no competing interests.

## Authors’ contributions

DB designed the whole study, performed the LH-PCR and statistical analyses, and drafted the manuscript. PC performed and interpreted the real-time PCR analyses. FQ helped with samples collection and DNA extraction, and contributed to manuscript revision and results discussion. PAB coordinated the work and helped to draft the manuscript. All authors read and approved the final manuscript.

## Supplementary Material

Additional file 1 Estimated marginal means of 16S rDNA molecules for month and for sanitary status based on real-time data analyzed by Sidak test. Click here for file
