# Supplementary material for: Endophytic bacterial community of grapevine leaves influenced by sampling date and phytoplasma infection process
Source: BMC Microbiol. 2014 Jul 21;14:198. doi: 10.1186/1471-2180-14-198 (PMC4223760; doi:10.1186/1471-2180-14-198)
Supplement: Additional file 1 — Estimated marginal means of 16S rDNA molecules for month and for sanitary status based on real-time data analyzed by Sidak test. [file 1471-2180-14-198-S1.docx]

Additional file 1

| **Multiple Comparisons** | | | | | | | |
| --- | --- | --- | --- | --- | --- | --- | --- |
| Dependent variable: 16S rDNA molecules | | | | | | | |
| **Month** | **(I) status** | **(J) status** | **Mean difference (I-J)** | **standard Error** | **Sig.^a^** | **Confidence intervals 95%^a^** | |
|  |  |  |  |  |  | **Lower bound** | **Upper bound** |
| 6 | S | I | 128,667 | 1119,478 | ,999 | -2652,476 | 2909,809 |
|  |  | R | 315,917 | 1119,478 | ,989 | -2465,226 | 3097,059 |
|  | I | S | -128,667 | 1119,478 | ,999 | -2909,809 | 2652,476 |
|  |  | R | 187,250 | 1036,436 | ,997 | -2387,588 | 2762,088 |
|  | R | S | -315,917 | 1119,478 | ,989 | -3097,059 | 2465,226 |
|  |  | I | -187,250 | 1036,436 | ,997 | -2762,088 | 2387,588 |
| 7 | S | I | -660,500 | 1036,436 | ,894 | -3235,338 | 1914,338 |
|  |  | R | -941,500 | 1119,478 | ,789 | -3722,643 | 1839,643 |
|  | I | S | 660,500 | 1036,436 | ,894 | -1914,338 | 3235,338 |
|  |  | R | -281,000 | 1119,478 | ,992 | -3062,143 | 2500,143 |
|  | R | S | 941,500 | 1119,478 | ,789 | -1839,643 | 3722,643 |
|  |  | I | 281,000 | 1119,478 | ,992 | -2500,143 | 3062,143 |
| 8 | S | I | -376,000 | 1036,436 | ,978 | -2950,838 | 2198,838 |
|  |  | R | -604,250 | 1036,436 | ,917 | -3179,088 | 1970,588 |
|  | I | S | 376,000 | 1036,436 | ,978 | -2198,838 | 2950,838 |
|  |  | R | -228,250 | 1036,436 | ,995 | -2803,088 | 2346,588 |
|  | R | S | 604,250 | 1036,436 | ,917 | -1970,588 | 3179,088 |
|  |  | I | 228,250 | 1036,436 | ,995 | -2346,588 | 2803,088 |
| 9 | S | I | -237,250 | 1036,436 | ,994 | -2812,088 | 2337,588 |
|  |  | R | -4080,250^*^ | 1036,436 | ,001 | -6655,088 | -1505,412 |
|  | I | S | 237,250 | 1036,436 | ,994 | -2337,588 | 2812,088 |
|  |  | R | -3843,000^*^ | 1036,436 | ,002 | -6417,838 | -1268,162 |
|  | R | S | 4080,250^*^ | 1036,436 | ,001 | 1505,412 | 6655,088 |
|  |  | I | 3843,000^*^ | 1036,436 | ,002 | 1268,162 | 6417,838 |
| 10 | S | I | 184,500 | 1036,436 | ,997 | -2390,338 | 2759,338 |
|  |  | R | 2108,000 | 1036,436 | ,138 | -466,838 | 4682,838 |
|  | I | S | -184,500 | 1036,436 | ,997 | -2759,338 | 2390,338 |
|  |  | R | 1923,500 | 1036,436 | ,197 | -651,338 | 4498,338 |
|  | R | S | -2108,000 | 1036,436 | ,138 | -4682,838 | 466,838 |
|  |  | I | -1923,500 | 1036,436 | ,197 | -4498,338 | 651,338 |
| Based on estimated marginal mean | | | | | | | |
| 1. Sidak correction.   *. The mean difference is significant at the .05 level.  6= June; 7= July; 8= August; 9= September; 10= October;  S=Healthy; I= Diseased; R= Recovered | | | | | | | |

| **Multiple comparisons** | | | | | | | | | | | | | | | |
| --- | --- | --- | --- | --- | --- | --- | --- | --- | --- | --- | --- | --- | --- | --- | --- |
| Dependent variable: 16S rDNA molecules | | | | | | | | | | | | | | | |
| **Month** | | **(I) status** | **(J) status** | | **Mean difference (I-J)** | | **standard Error** | | **Sig.^a^** | | **Confidence intervals 95%^a^** | | | |  |
|  |  |  |  |  |  |  |  |  |  |  | **Lower bound** | | **Upper bound** | |  |
| S | 6 | | | 7 | | 515,167 | | 1119,478 | | 1,000 | | -2787,972 | | 3818,306 | |
|  |  |  |  | 8 | | 104,667 | | 1119,478 | | 1,000 | | -3198,472 | | 3407,806 | |
|  |  |  |  | 9 | | -443,833 | | 1119,478 | | 1,000 | | -3746,972 | | 2859,306 | |
|  |  |  |  | 10 | | -2452,333 | | 1119,478 | | ,292 | | -5755,472 | | 850,806 | |
|  | 7 | | | 6 | | -515,167 | | 1119,478 | | 1,000 | | -3818,306 | | 2787,972 | |
|  |  |  |  | 8 | | -410,500 | | 1036,436 | | 1,000 | | -3468,613 | | 2647,613 | |
|  |  |  |  | 9 | | -959,000 | | 1036,436 | | ,988 | | -4017,113 | | 2099,113 | |
|  |  |  |  | 10 | | -2967,500 | | 1036,436 | | ,063 | | -6025,613 | | 90,613 | |
|  | 8 | | | 6 | | -104,667 | | 1119,478 | | 1,000 | | -3407,806 | | 3198,472 | |
|  |  |  |  | 7 | | 410,500 | | 1036,436 | | 1,000 | | -2647,613 | | 3468,613 | |
|  |  |  |  | 9 | | -548,500 | | 1036,436 | | 1,000 | | -3606,613 | | 2509,613 | |
|  |  |  |  | 10 | | -2557,000 | | 1036,436 | | ,163 | | -5615,113 | | 501,113 | |
|  | 9 | | | 6 | | 443,833 | | 1119,478 | | 1,000 | | -2859,306 | | 3746,972 | |
|  |  |  |  | 7 | | 959,000 | | 1036,436 | | ,988 | | -2099,113 | | 4017,113 | |
|  |  |  |  | 8 | | 548,500 | | 1036,436 | | 1,000 | | -2509,613 | | 3606,613 | |
|  |  |  |  | 10 | | -2008,500 | | 1036,436 | | ,457 | | -5066,613 | | 1049,613 | |
|  | 10 | | | 6 | | 2452,333 | | 1119,478 | | ,292 | | -850,806 | | 5755,472 | |
|  |  |  |  | 7 | | 2967,500 | | 1036,436 | | ,063 | | -90,613 | | 6025,613 | |
|  |  |  |  | 8 | | 2557,000 | | 1036,436 | | ,163 | | -501,113 | | 5615,113 | |
|  |  |  |  | 9 | | 2008,500 | | 1036,436 | | ,457 | | -1049,613 | | 5066,613 | |
| I | 6 | | | 7 | | -274,000 | | 1036,436 | | 1,000 | | -3332,113 | | 2784,113 | |
|  |  |  |  | 8 | | -400,000 | | 1036,436 | | 1,000 | | -3458,113 | | 2658,113 | |
|  |  |  |  | 9 | | -809,750 | | 1036,436 | | ,997 | | -3867,863 | | 2248,363 | |
|  |  |  |  | 10 | | -2396,500 | | 1036,436 | | ,229 | | -5454,613 | | 661,613 | |
|  | 7 | | | 6 | | 274,000 | | 1036,436 | | 1,000 | | -2784,113 | | 3332,113 | |
|  |  |  |  | 8 | | -126,000 | | 1036,436 | | 1,000 | | -3184,113 | | 2932,113 | |
|  |  |  |  | 9 | | -535,750 | | 1036,436 | | 1,000 | | -3593,863 | | 2522,363 | |
|  |  |  |  | 10 | | -2122,500 | | 1036,436 | | ,380 | | -5180,613 | | 935,613 | |
|  | 8 | | | 6 | | 400,000 | | 1036,436 | | 1,000 | | -2658,113 | | 3458,113 | |
|  |  |  |  | 7 | | 126,000 | | 1036,436 | | 1,000 | | -2932,113 | | 3184,113 | |
|  |  |  |  | 9 | | -409,750 | | 1036,436 | | 1,000 | | -3467,863 | | 2648,363 | |
|  |  |  |  | 10 | | -1996,500 | | 1036,436 | | ,465 | | -5054,613 | | 1061,613 | |
|  | 9 | | | 6 | | 809,750 | | 1036,436 | | ,997 | | -2248,363 | | 3867,863 | |
|  |  |  |  | 7 | | 535,750 | | 1036,436 | | 1,000 | | -2522,363 | | 3593,863 | |
|  |  |  |  | 8 | | 409,750 | | 1036,436 | | 1,000 | | -2648,363 | | 3467,863 | |
|  |  |  |  | 10 | | -1586,750 | | 1036,436 | | ,760 | | -4644,863 | | 1471,363 | |
|  | 10 | | | 6 | | 2396,500 | | 1036,436 | | ,229 | | -661,613 | | 5454,613 | |
|  |  |  |  | 7 | | 2122,500 | | 1036,436 | | ,380 | | -935,613 | | 5180,613 | |
|  |  |  |  | 8 | | 1996,500 | | 1036,436 | | ,465 | | -1061,613 | | 5054,613 | |
|  |  |  |  | 9 | | 1586,750 | | 1036,436 | | ,760 | | -1471,363 | | 4644,863 | |
| R | 6 | | | 7 | | -742,250 | | 1119,478 | | ,999 | | -4045,389 | | 2560,889 | |
|  |  |  |  | 8 | | -815,500 | | 1036,436 | | ,997 | | -3873,613 | | 2242,613 | |
|  |  |  |  | 9 | | -4840,000^*^ | | 1036,436 | | ,000 | | -7898,113 | | -1781,887 | |
|  |  |  |  | 10 | | -660,250 | | 1036,436 | | ,999 | | -3718,363 | | 2397,863 | |
|  | 7 | | | 6 | | 742,250 | | 1119,478 | | ,999 | | -2560,889 | | 4045,389 | |
|  |  |  |  | 8 | | -73,250 | | 1119,478 | | 1,000 | | -3376,389 | | 3229,889 | |
|  |  |  |  | 9 | | -4097,750^*^ | | 1119,478 | | ,007 | | -7400,889 | | -794,611 | |
|  |  |  |  | 10 | | 82,000 | | 1119,478 | | 1,000 | | -3221,139 | | 3385,139 | |
|  | 8 | | | 6 | | 815,500 | | 1036,436 | | ,997 | | -2242,613 | | 3873,613 | |
|  |  |  |  | 7 | | 73,250 | | 1119,478 | | 1,000 | | -3229,889 | | 3376,389 | |
|  |  |  |  | 9 | | -4024,500^*^ | | 1036,436 | | ,003 | | -7082,613 | | -966,387 | |
|  |  |  |  | 10 | | 155,250 | | 1036,436 | | 1,000 | | -2902,863 | | 3213,363 | |
|  | 9 | | | 6 | | 4840,000^*^ | | 1036,436 | | ,000 | | 1781,887 | | 7898,113 | |
|  |  |  |  | 7 | | 4097,750^*^ | | 1119,478 | | ,007 | | 794,611 | | 7400,889 | |
|  |  |  |  | 8 | | 4024,500^*^ | | 1036,436 | | ,003 | | 966,387 | | 7082,613 | |
|  |  |  |  | 10 | | 4179,750^*^ | | 1036,436 | | ,002 | | 1121,637 | | 7237,863 | |
|  | 10 | | | 6 | | 660,250 | | 1036,436 | | ,999 | | -2397,863 | | 3718,363 | |
|  |  |  |  | 7 | | -82,000 | | 1119,478 | | 1,000 | | -3385,139 | | 3221,139 | |
|  |  |  |  | 8 | | -155,250 | | 1036,436 | | 1,000 | | -3213,363 | | 2902,863 | |
|  |  |  |  | 9 | | -4179,750^*^ | | 1036,436 | | ,002 | | -7237,863 | | -1121,637 | |
| Based on estimated marginal mean | | | | | | | | | | | | | | | |
| 1. Sidak correction.   *. The mean difference is significant at the .05 level.  6= June; 7= July; 8= August; 9= September; 10= October;  S=Healthy; I= Diseased; R= Recovered | | | | | | | | | | | | | | | |
